# Genomic insights into *Enterococcus faecium* isolates from marine bivalves highlight One Health concerns and healthcare linkages

**DOI:** 10.1099/mgen.0.001154

**Published:** 2023-12-12

**Authors:** Amalie von Barner Tvedegaard Heim, Jessin Janice, Jørgen Vildershøj Bjørnholt, Bjørn Tore Lunestad, Kristin Hegstad, Cecilie Smith Svanevik

**Affiliations:** ^1^​ Institute of Marine Research (IMR), Post box 1870, Nordnes, Bergen, Norway; ^2^​ Norwegian National Advisory Unit on Detection of Antimicrobial Resistance, University Hospital of North Norway (UNN), N-9038 Tromsø, Norway; ^3^​ Department of Clinical Microbiology, Oslo University Hospital, Oslo, Norway. PO box 4950 Nydalen 0424 Oslo, Norway; ^4^​ Institute of Clinical Medicine, University of Oslo (UiO), Oslo, Norway. PO box 1171 - Blindern, 0318 Oslo, Norway; ^5^​ Research Group for Host-Microbe Interactions, UiT the Arctic University of Norway, PO box 6050 Langnes, N-9037 Tromsø, Norway

**Keywords:** antimicrobial resistance, enterococci, feacal indicator, global phylogeny, marine environment

## Abstract

Enterococci, especially *Enterococcus faecium *, are one of today’s leading causes of multidrug-resistant infections in hospital settings. The marine environment may harbour enterococci, but its role as an evolutionary niche and as a vector for the spread of enterococci is sparsely investigated. Hence, by applying enterococci in bivalves as a sentinel tool, this study aimed to describe the prevalence of enterocooci along the Norwegian coast and in addition the phylogeny of *E. faecium* in particular. Enterococci in batch samples of marine bivalves, harvested from 86 different locations, were quantitatively examined by a culture-dependent most probable number (MPN) method. Isolates were identified by MALDI-TOF-MS prior to antimicrobial susceptibility testing by broth microdilution. In-detail analyses of a representative selection of *E. faecium* isolates (*n*=148) were done by Illumina whole-genome sequencing, and assembled genomes were compared to closed *E. faecium* genomes in the public databases and to genomes from commensal and clinical isolates from Norway. Diversity among *E. faecium* within the same batch sample of bivalves was also explored. Enterococci were detected in 287 of the 471 examined bivalve samples, but in low concentrations with a median value of <18 MPN /100 g. From positive samples, 479 isolates of enterococci were identified belonging to ten different species, where *E. faecium* (*n*=247), *Enterococcus hirae* (*n*=114) and *Enterococcus faecalis* (*n*=66) were most frequently found. Resistance towards one or more antimicrobial agents was observed in 197 isolates (41 %), none of the isolates showed acquired resistance to vancomycin or linezolid. Phylogenetic analyses revealed high diversity among the *E. faecium* isolates and showed that the marine niche is dominated by strains from the non-clinical setting belonging to clade A2 (*n*=85) and B (* E. lactis *) (*n*=60). Only three isolates belonged to the hospital-associated clade A1 (ST80 and ST117). Two of these clustered with one isolate from a hospitalized patient and one from a non-hospitalized person. This study demonstrated a high prevalence, but low concentrations of enterococci in bivalves, and low levels of antimicrobial resistance. *E. faecium* genomes showed high population diversity and that very few *E. faecium* isolates in bivalves may have arisen from the human healthcare system. A systematic surveillance of target micro-organisms applying methods examining multiple isolates from the same bivalve sample provides important data to assess the enterococcal phylogeny, antimicrobial resistance and the level of faecal pollution in the marine environment.

## Data Summary

All the *E. faecium* genomes were short-read sequenced. The sequenced genomes were submitted to NCBI under the project PRJNA990824. Metadata and the accession numbers of the genomes are presented in Table S6, available in the online version of this article. The authors confirm all the supporting data, code and protocols have been provided within the article or through supplementary data files.

**Impact Statement**

This study describes the prevalence of enterococci in marine bivalves along the Norwegian coast. Importantly, the 148 analysed E. faecium genomes contribute to reduce the knowledge gap on this species from the marine environment. Genomic databases are dominated by clinical strains and hence, this work provides data needed to improve our phylogenetic understanding of E. faecium outside the clinical settings. The data on antimicrobial resistance among enterococci provides baseline data from the marine environment important for future monitoring of possible changes. Furthermore, this study demonstrates that the chosen approach for obtaining isolates enabled insight into inter- and intra-species diversity within the same sample.

## Introduction

Enterococci are ancient Gram-positive bacteria belonging to the genus *Enterococcus* and naturally found in the gastrointestinal (GI) tract of humans and animals, including insects [1]. The World Health Organization is regarding antimicrobial resistance in bacteria as one of the major threats to the global public health [2], and the development of pathogenic, multidrug-resistant enterococci is of great concern in hospitals [3, 4].

It is suggested that the enterococci arose from aquatic ancestors during the terrestrialization 425 million years ago [5], and hence the genus is not recognized to be indigenous in the sea. Enterococci may enter the marine environment via sewage from the community, including healthcare facilities, and run-offs from land, and are frequently reported in faecal-contaminated coastal environments [6]. When shed into the sea, their fate and prevalence are dependent on several biotic and abiotic factors, such as sunlight, temperature, salinity, available nutrients and predator density [7, 8]. However, Enterococci are known to be sturdy and capable of adapting to and surviving in a variety of these environmental stress factors [9].

Marine bivalves are filter feeders and retain particles including bacteria and viruses present in their surrounding waters and are important in bioremediation along the coast. Because of their accumulating ability, marine bivalves are frequently used as indicators for microbial contamination [10]. Their water clearance rate depends on factors such as size, habitat and available feed organisms, and an adult blue mussel (*Mytilus edulis*) can clear between 12 and 240 l (mean 72) of water per day [11]. Marine bivalves are also important seafood organisms harvested and cultured along the coast. To protect consumer’s health and ensure food safety, surveillance programmes are enforced according to EU regulations [12], which set criteria for approval of bivalve production areas based on the concentration of the faecal contaminant indicator *Escherichia coli* analysed by a standardized method (ISO 16649–3, 2005). Bivalves from class A areas are approved for direct consumption, whereas class B and class C areas would need depuration and/or heat treatment prior to entering the market.

Currently, 57 valid enterococcal species are known [13] of which *Enterococcus faecium* and *E. faecalis* are the species most frequently involved in human infections [14]. *E. faecium* isolates show large genomic diversity and can be divided phylogenetically into clade A1, A2 and B. Clade A1 represents hospital-associated strains involved in infection, clade A2 strains are isolated from humans in the community, livestock and domestic animals, while clade B also contains human commensal strains. Strains from clades A2 and B are rarely related to human disease [5, 15], and clade B has been reclassified to *E. lactis* [16]. There is currently a knowledge gap about the phylogeny of enterococci found in the marine environment, as available whole genomes in public databases are limited and only few isolates belong to *E. faecium* [17–21].

In 2015, vancomycin-resistant *E. faecalis* and *E. faecium* (VRE) represented the eighth most frequent cause of infections by antimicrobial resistant bacteria in Europe [22]. In Norway, *Enterococcus* is the fifth most common bacterial genus found in blood culture isolates [23], however, the prevalence of VRE is low. In 2019, 204 VRE were registered and in 2020 only 75, comprising mostly *E. faecium* with the *vanB* genotype. All these vancomycin-resistant *E. faecium* isolates belonged to well-known hospital-adapted clones that have been reported also from other countries [24].

The main objective of this study was to provide novel knowledge about enterococci in a One Health perspective that could help curb the development of antimicrobial resistance. Specifically, we aimed to document the prevalence of *Enterococcus* spp. in the marine environment using marine bivalves as sentinel tools anddescribe the prevalences of antimicrobial resistance. We further aimed toincrease the knowledge of the phylogeny of *E. faecium* from the marineenvironment based on whole genome sequences.

## Methods

### Sampling and study site

**Fig. 1.**
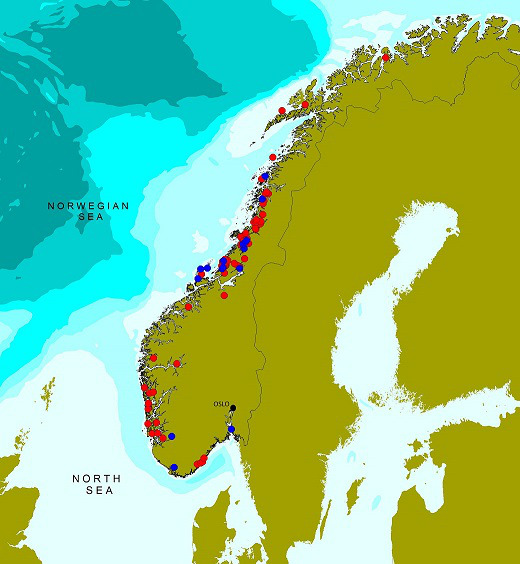
Overview of the 86 sampling areas along the Norwegian coast for samples collected in 2016, 2019 and 2020. The red dots are locations with positive samples (70), whereas the blue dots are locations with negative samples (16).

**Fig. 2.**
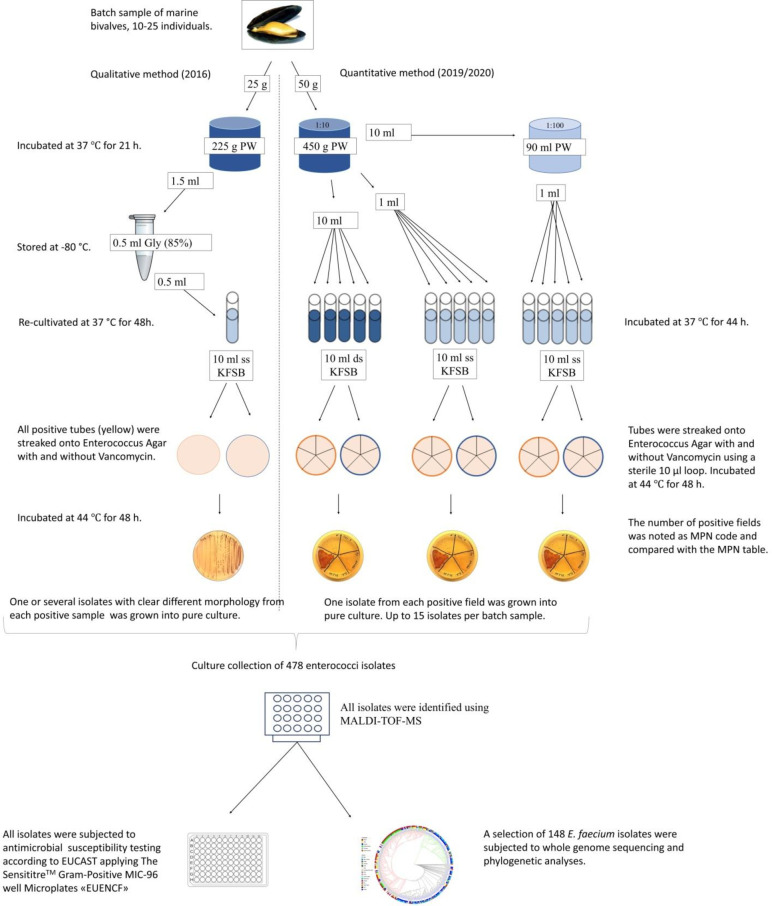
Schematic overview of applied methods for qualitative and quantitative detection of enterococci isolates, antimicrobial susceptibility testing and phylogenetic analyses. PW = peptone water, Gly = glycerol, ssKFSB = single strength KF Streptococcus Broth. dsKFSB = double strength Streptococcus Broth.

Sampling was coordinated with the Norwegian Food Safety Authority (NFSA) and their annually ongoing surveillance programme for bivalves [25] conducted by the Institute of Marine Research (IMR). In total, 471 batch samples from 2016, 2019 and 2020 were collected from 86 locations. Among these, 462 were collected by the NFSA from 81 locations, mainly comprising samples from commercial harvest areas (either wild population from natural habitats, or bivalves settled on rope systems), and final product control samples. These samples were sent to the laboratory and analysed within 24 h after sampling. In addition, nine samples were collected by IMR at five locations not included in the NFSA surveillance programme. These samples were treated in the same way as samples from NFSA. The origin of samples is found in Table S1. Each sample consisted of batches of bivalves and the complete collection comprised 389 samples of blue mussels (*Mytilus edulis*), 44 samples of European flat oysters (*Ostrea edulis*), 27 samples of great scallops (*Pecten maximus*), four samples of horse mussels (*Modiolus modiolus*), three samples of ocean quahogs (*Arctica islandica*), two samples of pullet carpet shells (*Venerupis corrugata*) and one sample each of pacific oysters (*Magallana gigas*) and cockles (fam. *Cardiidae*). Fig. 1 shows the distribution of sampling areas. All samples were examined for the concentration of *E. coli* based on a most probable number (MPN) method according to ISO 16649–3 (2005) [26]. Further analyses are described in detail below and presented schematically in Fig. 2.

### Qualitative analysis of enterococci

The bivalve batch samples collected in 2016 (*n*=244) were only analysed quantitatively. Soft material and intravalvular fluid from 10 to 25 living bivalve individuals were first homogenized in a Stomacher (Interscience) at 185 r.p.m. for 2.5 min. From the homogenate, 25 g was diluted 1 : 10 with buffered peptone water (bioMérieux) further homogenized for 30 s, prior to enrichment at 37 °C±1 °C for 21±3 h. Aliquots of 1.5 ml from the enriched homogenate were mixed with 0.5 ml glycerol (85 %) prior to storage at −80 °C [27]. From the frozen homogenates, 0.5 ml was re-cultivated in 10 ml Streptococcus Broth at 37±1 °C for 48±2 h, and subsequently 10 µl was streaked on Enterococcus Agar (BD DifcoTM) and incubated in water bath at 44±1 °C for 48±2 h. One or several isolates in case of clear difference in morphology, were picked and grown into pure culture. See protocol overview in Fig. 2.

### Quantitative analyses of enterococci

Among the 2019 and 2020 samples (*n*=227), 218 were quantitatively analysed for enterococci according to the five-times-three dilution with the MPN method APHA 2001 for enterococci and faecal streptococci in foods [28, 29]. Preparation of the homogenate was done similarly as for the qualitative method. From the homogenate, 50 g were diluted 1 : 10 with buffered peptone water and subsequently a 1 : 100 dilution was prepared. From the 1 : 10 dilution, 10 ml and 1 ml were transferred to the first and second set of five parallel tubes of containing double and single strength of KF Streptococcus Broth (BD Difco), respectively. From the 1 : 100 dilution, 1 ml was transferred to a third set of five tubes with the same broth. This would yield a final amount of 1, 0.1 and 0.01 g sample material in each dilution set of tubes. All tubes were incubated at 37±1 °C for 48±2 h. Enterococci-positive tubes changed colour from red to yellow and were confirmed as presumptive enterococci (pink and purple colonies) after streaking of 10 µl on Enterococcus Agar (BD Difco) and incubation in water bath at 44±1 °C for 48±2 h [30]. The number of positive plates from each dilution was registered as the MPN code and the concentration was obtained from a standardized MPN table [31]. The limit of quantification (LOQ) of this method is 18 MPN/100 g. From each positive plate, one isolated colony was picked and grown into pure culture. Depending on the number of positive plates from each sample, 0–15 isolates could be collected from each sample. The remaining nine samples were only analysed qualitatively as described above. See protocol overview in Fig. 2.

### Species identification

All obtained isolates were identified by MALDI-TOF MS according to protocol from manufacturer (BRUKER). The applied BRUKER library [MALDI Biotyper Compass Explorer (v. 2020)] contains 34 *Enterococcus* species but is unable to distinguish *E. faecium* from *E. lactis* [32].

### Control strains

The *E. faecalis* strain CCUG 9997 was used as a positive control and *E. coli* CCUG 17620 as a negative control for all analysis.

### Antimicrobial susceptibility testing

The antimicrobial susceptibility testing was done according to International Standard ISO 20776–1 (2019) applying broth microdillution. The Sensititre Gram-Positive MIC 96-well Microplates ‘EUENCF’ (Thermo Fischer) were used, and MIC values were determined manually in the Sensititre SWIN Software System (Thermo Fischer). Susceptibility categorization was based on European Committee on Antimicrobial Susceptibility Testing breakpoints for *Enterococcus* spp. [33].

### Whole-genome sequencing

For genotypic analyses, 148 *E. faecium* were selected for whole-genome sequencing at Oslo University Hospital and the Genomics Support Centre Tromsø (UiT – the Arctic University of Norway). Three isolates were selected for WGS based on their resistance profile, and a random selection of 131 were included stratified per year and county. The WGS collection was further extended with 14 isolates to examine the diversity among *E. faecium* within a sample. Sequencing was done by Illumina with adapter removal and quality trimming of the raw reads performed by trimmomatic v0.39 [34]. Genome assembly was done using SPAdes v3.13.0 [35] and the quality of assembled genomes was assessed using quast v5.0.2 [36]. A cut-off maximum of 400 contigs and minimum 40×genome coverage was used to consider the assemblies as eligible to be included in the analyses. Moreover, the genome size should not show more than 10 % fluctuation compared to the smallest and biggest complete *E. faecium* genome assemblies in the National Center for Biotechnology Information (NCBI) database. Antimicrobial resistance genes were identified *in silico* from the assemblies using the NCBI bacterial AMR reference gene database (PRJNA313047) [37] in ABRicate tool [38] v0.8.7. Replicon types were predicted using PlasmidFinder database in ABRicate tool [38] v0.8.7.

### Construction of phylogenetic trees

To explore the phylogenetic relationship between the *E. faecium* from bivalves and publicly available genome sequences on NCBI, a global phylogenetic tree was generated based on the core genomes. Closed genomes of *E. faecium* (*n*=239) from NCBI were retrieved for the global tree. A core-genome tree for only marine *E. faecium* was constructed to display metadata including genome information. Interactive Tree Of Life (iTOL) was applied to display metadata in the trees [39]. Phylogenetic trees were constructed using Parsnp v1.2 [40]. Multilocus sequence typing (MLST) was performed using MLST tool v2.11 [41]. For high-resolution typing, Minimum Spanning Tree (MST) was generated based on the 1423 core genes of *E. faecium* scheme of SeqSphere+software v6.0.2 [42]. We used the default ≤20 allelic differences as a threshold for cluster calculation and clonal relatedness of *E. faecium* [43]. Comparisons were made between the marine *E. faecium* genomes and *E. faecium* genomes from Norwegian clinical and human population samples collected at the Norwegian National Advisory Unit on Detection of Antimicrobial Resistance.

## Results

### Significant prevalence, but low concentrations of enterococci

**Fig. 3.**
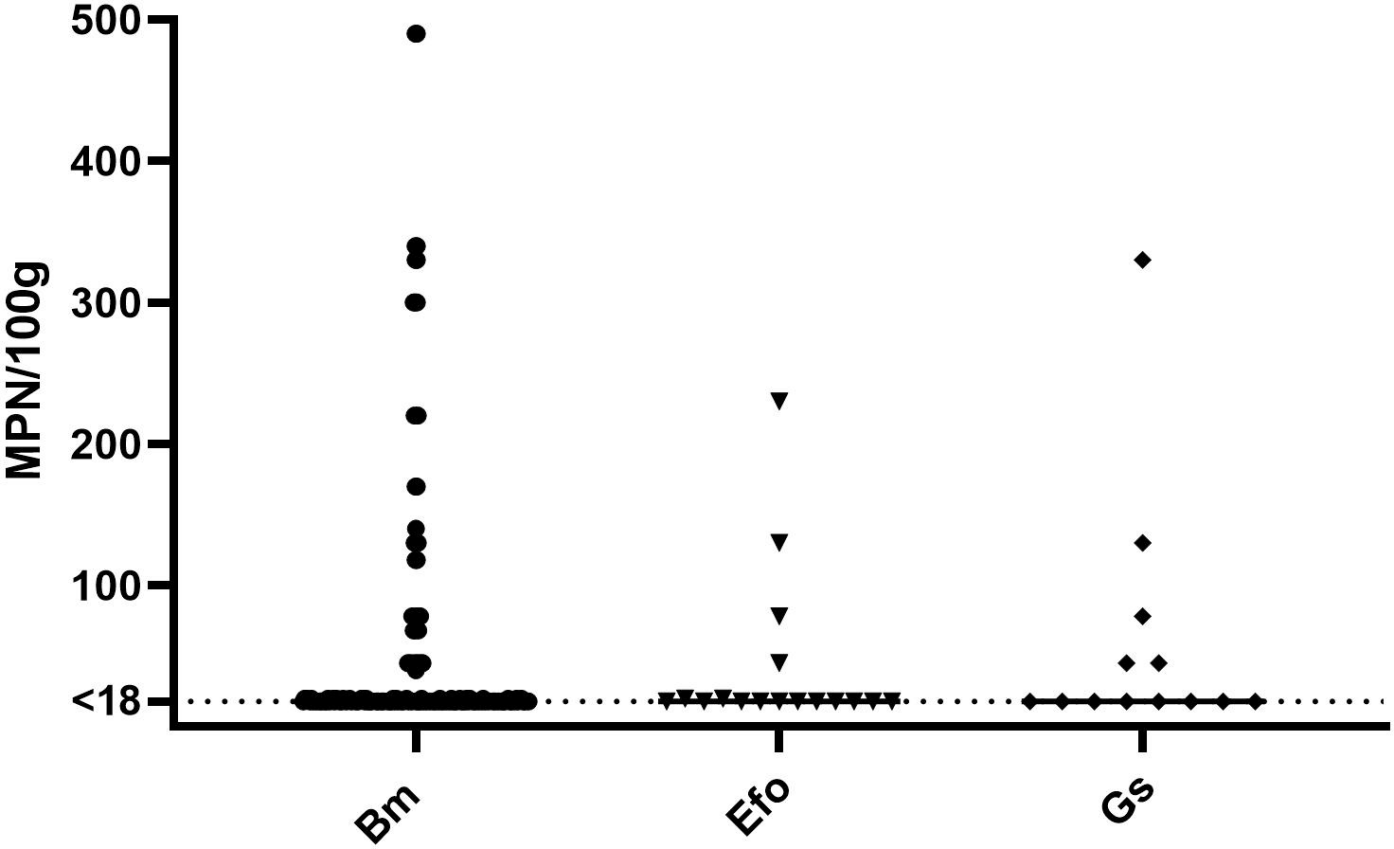
Most probable number (MPN) of Enterococci per 100 g sample from batches of Bm=blue mussels (*n*=411), Efo=European flat oysters (*n*=40) and Gs=Great scallops (*n*=23). Limit Of Quantification (LOQ)=18 MPN/100g. *Five values of 790, 1300, 1300, 2000 and 3500 MPN/100g exceeds the range of the axis.

Among all the examined 471 marine bivalve samples, 60.7 % (95 % CI; 56.2–65.2) (*n*=286) contained enterococci. The quantitative analysis applied for samples collected in 2019/2020 found a lower prevalence of 43.2 % (95 % CI; 36.6–49.8) compared to the qualitative analysis used in 2016 where 77.1 % (95 % CI; 71.6–82.6) was found. The positive samples originated from 70 different locations, where the frequency of positive samples were Agder (4.9 %), Rogaland (8.4 %), Vestland (16.4 %), Trøndelag (51.0 %), Nordland (16.4 %) and Troms (2.8 %). The quantitative analyses (*n*=218) showed that the median enterococci value was <18 MPN/100 g for all bivalve species (Table S1). The highest detected values were 3500 MPN/100 g for blue mussels, 330 MPN/100 g for great scallops and 230 MPN/100 g for European flat oysters (Fig. 3). In the one batch sample with cockles, 20 MPN/100 g enterococci were detected. No quantifiable enterococci were found in carpet shells, ocean quahogs or pacific oysters.

### Ten different enterococcal species found in marine bivalves

The total isolate collection deriving from these 286 positive samples comprised 479 enterococci where ten different species were identified by MALDI-TOF-MS, with *E. faecium *, *E. hirae* and *E. faecalis* being the most frequent (Table 1).

**Table 1.** Overview of the 479 isolates identified with MALDI-TOF MS, which species they belonged to and from which bivalve species they originated

Marine bivalve species							
*** Enterococcus* spp**.	**Blue mussels**	**European flat oysters**	**Great scallops**	**Horse mussels**	**Pullet carpet shells**	**Cockles**	**Total**
E. faecium*	206	24	15	1	1		247
*** E. hirae ***	101	5	6	1		1	114
*** E. faecalis ***	53	11	2				66
*** E. durans ***	25						25
*** E. casseliflavus ***	12						12
*** E. avium ***	3			1			4
*** E. thailandicus ***	4						4
*** E. gallinarum ***	3						3
*** E. mundtii ***	3						3
*** E. villorum ***	1				–		1
**Total**	411	40	23	3	1	1	479

* *E. faecium* clade B has been reclassified to *E. lactis* [16]. Among the 148 sequenced *E. faecium* genomes, 40.5 % isolates were *E. lactis*.

### Phenotypic antimicrobial resistance in enterococci and *E. faecium*

Antimicrobial susceptibility testing showed that 41.0 % (*n*=197) of the 479 isolates expressed resistance to one or more antimicrobial agents, with the highest prevalence in *E. faecium* with 71 % (*n*=176) among the 247 isolates. Resistance profiles for all isolates could be found in Table S2. Resistance in *E. faecium* isolates was seen towards 11 different antimicrobials. Three of these isolates originating from blue mussels were resistant to aminopenicillins, quinolones and imipenem, and two conferred in addition high level resistance to gentamicin and streptomycin. One isolate, also originating from blue mussels, was resistant to quinolones, quinupristin/dalfopristin and tigecycline. Another isolate, originating from a batch sample of great scallops, was resistant to streptomycin, imipenem and quinupristin/dalfopristin. None of the isolates had acquired resistance towards vancomycin or linezolid. The MIC distribution for *E. faecium* is found in Table S3.

### Genomic diversity of *E. faecium*

**Fig. 4.**
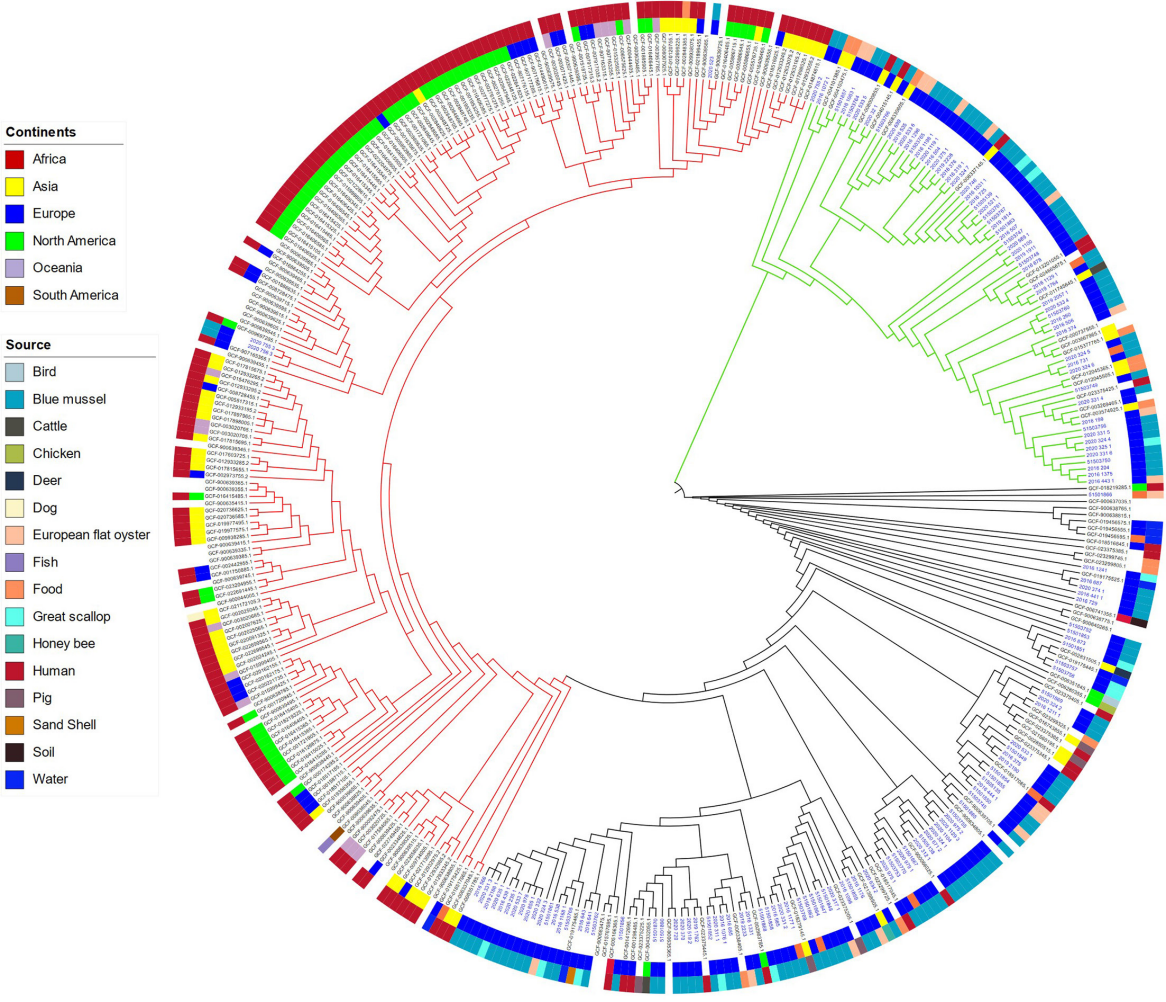
Parsnp tree of the 148 *E. faecium* from marine bivalves (names coloured blue) and 239 closed *E. faecium* genomes downloaded from NCBI (coloured black). Branches of the tree are coloured to highlight clade B (green, *E. lactis *), hospital-associated clade A1 (red) and A2 (black). Metadata added from the inner layer are continents and isolation source.

**Fig. 5.**
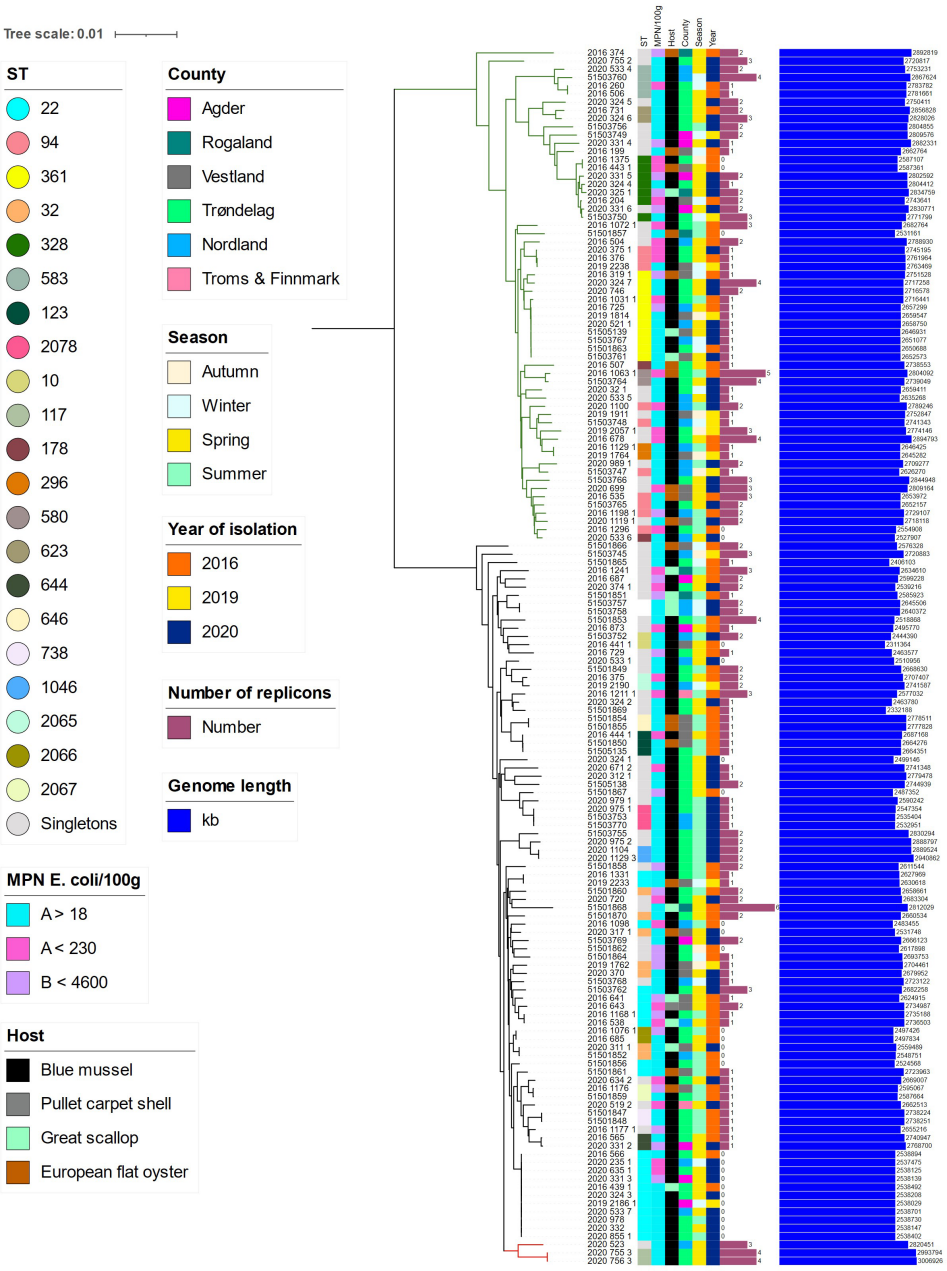
Core genome Parsnp tree of 148 *E. faecium* from marine bivalves with added metadata as indicated by legends. Branches of the tree are coloured to highlight clade B (green, *E. lactis *), hospital-associated clade A1 (red) and A2 (black).

Genomic analyses of the 148 selected *E. faecium* revealed that 88 isolates belonged to the *E. faecium* clade A and 60 isolates to clade B (* E. lactis *). The three ampicillin-resistant isolates, two of them also high-level gentamicin resistant, clustered with human isolates in the hospital-associated clade A1 in the global tree of *E. faecium* (Fig. 4) and belonged to sequence types ST80 (*n*=1) and ST117 (*n*=2), which are among the dominant STs also in Norwegian hospitals [24]. These three isolates originated from batch samples of bivalves with relatively low concentrations of enterococci (20, 68 and 68 MPN/100 g). The genomes showed broad sequence diversity with 75 different STs including 54 singletons. Strains showing the same ST and even those closest in the phylogenetic tree were often from different locations (more than one county) and timepoints (different season and years). Genome length varied between 2.3–3.0 Mb and number of plasmid replicons identified by *rep* typing varied from zero to six. The three genomes belonging to the hospital-associated subclade showed genome length 2.8–3.0 Mb and three to four replicons (Fig. 5; Table S4).

### Relatedness of *E. faecium* from marine and human samples

**Fig. 6.**
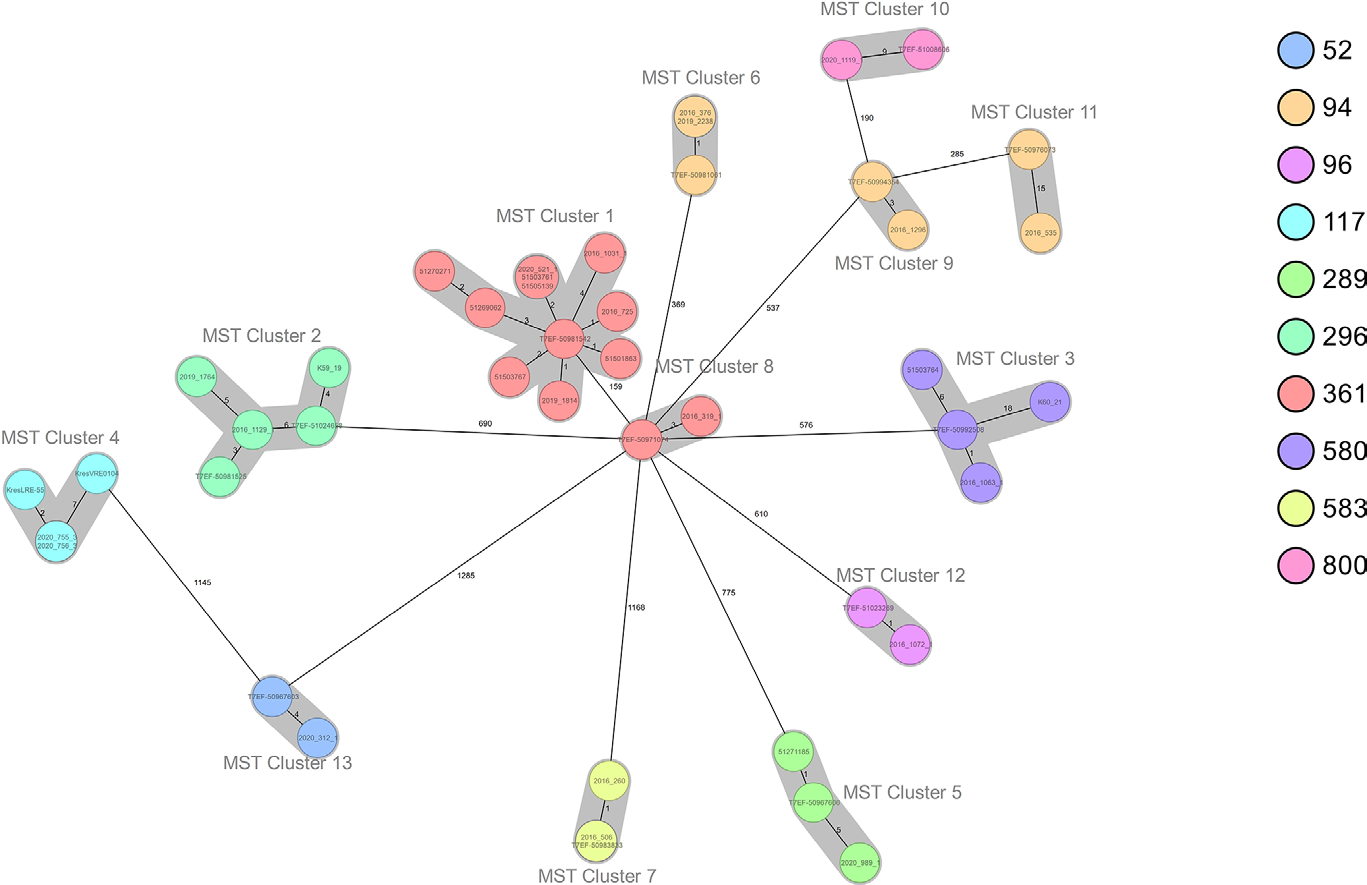
Minimum spanning tree (MST) built from core-genome allelic profile of Norwegian *E. faecium* isolates from different sources (marine, clinical and human population samples) using Ridom-SeqSphere+ software with the integrated core-genome (cg) MLST scheme with *E. faecium* Aus0004 as the reference strain. The isolates are colour coded according to sequence type. Genetically closely related isolates (≤20 allelic distances) are highlighted in grey.

Comparison by cgMLST of the marine *E. faecium* genomes to *E. faecium* genomes from Norwegian clinical and human population samples revealed 13 clusters containing *E. faecium* from both marine and human sources suggesting relatedness between marine and human *E. faecium *. Clusters with both marine and clinical isolates were detected for five different STs (ST117, ST289, ST296, ST361 and ST580) and with both marine and human population samples for nine different STs (ST52, ST94, ST96, ST289, ST296, ST361, ST580, ST583 and ST800) (Fig. 6 and Table 2). Most interestingly, the hospital-associated subclade ST117 cluster (MST cluster 4) consisted of four isolates from 2020 including two from farmed blue mussels from different locations in Trøndelag county harvested at the same day in May, one vancomycin-resistant clinical isolate also from Trøndelag county isolated in January and one linezolid-resistant clinical isolate from another county isolated in November. The two marine isolates showed no allelic differences and were most closely related to the linezolid-resistant isolate with only two allelic differences. Bivalve isolates also showed close relatedness to human population samples, i.e. in a cluster of three ST583 (MST cluster 7), one human population isolate from Troms County from 2015 showed no allelic differences to one marine isolate and one allelic difference to another marine isolate both from blue mussels harvested at two different locations in Trøndelag county in 2016 (Fig. 6, Tables 2 and S4). Please note that Trøndelag is the main bivalve production area in Norway, having both half of the sampled bivalves and half of the positive samples. Despite that the link between enterococci in bivalves and humans are valid, the overrepresentation of positive samples from Trøndelag could be a sampling bias, as similar coverage from other areas are missing.

**Table 2.** Characteristics of strains showing relatedness in Fig. 6. Norwegian clinical and human population samples were collected at the Norwegian National Advisory Unit on Detection of Antimicrobial Resistance

MST cluster	Strain ID	Source	Clade	ST	CT	Year	County	Isolation site	VAN^R^/LIN^R^
1	2016-261/1	Marine	B	361	1901	2016	Trøndelag	Blue mussels	No
	2020-311/3	Marine	B	361	1901	2020	Vestland	Great scallops	No
	2020-66/2	Marine	B	361	1901	2020	Nordland	Blue mussels	No
	2020-311/4	Marine	B	361	1901	2020	Vestland	Great scallops	No
	2016-1031/1	Marine	B	361	1901	2016	Trøndelag	Blue mussels	No
	2016-725/1	Marine	B	361	1901	2016	Nordland	Blue mussels	No
	2019-1814/1	Marine	B	361	1901	2019	Vestland	Blue mussels	No
	2020-521/1	Marine	B	361	1901	2020	Nordland	Blue mussels	No
	51 269 062	Hospitalized patient	B	361	1901	2014	Vestland	Blood	No
	51 270 271	Hospitalized patient	B	361	1901	2014	Trøndelag	Blood	No
	T7EF-50981542	Non-hospitalized person	B	361	1901	2015	Troms	Faeces	No
2	2016-1129/1	Marine	B	296	426	2016	Nordland	Blue mussels	No
	2019-1764/1	Marine	B	296	426	2019	Vestland	Blue mussels	No
	K59_19	Hospitalized patient	B	296	426	2008	Møre og Romsdal	Blood	No
	T7EF-50981525	Non-hospitalized person	B	296	426	2015	Troms	Faeces	No
	T7EF-51024658	Non-hospitalized person	B	296	426	2015	Troms	Faeces	No
3	2020-368/1	Marine	B	580	5402	2020	Trøndelag	Blue mussels	No
	2016-1063/1	Marine	B	580	5402	2016	Vestland	Blue mussels	No
	K60_21	Hospitalized patient	B	580	5402	2008	Østfold	Blood	No
	T7EF-50992508	Non-hospitalized person	B	580	5402	2015	Troms	Faeces	No
4	2020-755/3	Marine	A1	117	2505	2020	Trøndelag	Blue mussels	No
	2020-756/3	Marine	A1	117	2505	2020	Trøndelag	Blue mussels	No
	KresVRE0104	Hospitalized patient	A1	117	929	2020	Trøndelag	Urine	VAN^R^
	KresLRE-55	Hospitalized patient	A1	117	2505	2020	Oslo	Clinical site*	LIN^R^
5	2020-989/1	Marine	B	289	5243	2020	Nordland	Blue mussels	No
	51 271 185	Hospitalized patient	B	289	5243	2014	Vestfold	Blood	No
	T7EF-50967606	Non-hospitalized person	B	289	5243	2015	Troms	Faeces	No
6	2016-376/1	Marine	B	94	5277	2016	Trøndelag	Blue mussels	No
	2019-2238/1	Marine	B	94	5277	2019	Vestland	Blue mussels	No
	T7EF-50981061	Non-hospitalized person	B	94	5277	2015	Troms	Faeces	No
7	2016-260/1	Marine	B	583	5342	2016	Trøndelag	Blue mussels	No
	2016-506/1	Marine	B	583	5342	2016	Trøndelag	Blue mussels	No
	T7EF-50983833	Non-hospitalized person	B	583	5342	2015	Troms	Faeces	No
8	2016-319/1	Marine	B	361	5274	2016	Vestland	European flat oysters	No
	T7EF-50971074	Non-hospitalized person	B	361	5274	2015	Troms	Faeces	No
9	2016-1296/1	Marine	B	94	5414	2016	Trøndelag	Blue mussels	No
	T7EF-50994354	Non-hospitalized person	B	94	5414	2015	Troms	Faeces	No
10	2020-1119/1	Marine	B	800	5461	2020	Vestland	European flat oysters	No
	T7EF-51008606	Non-hospitalized person	B	800	5461	2015	Troms	Faeces	No
11	2016-535/1	Marine	B	94	5218	2016	Vestland	European flat oysters	No
	T7EF-50976073	Non-hospitalized person	B	94	5218	2015	Troms	Faeces	No
12	2016-1072/1	Marine	B	96	5300	2016	Trøndelag	Blue mussels	No
	T7EF-51023269	Non-hospitalized person	B	96	5300	2015	Troms	Faeces	No
13	2020-312/1	Marine	A2	52	2138	2020	Trøndelag	Blue mussels	No
	T7EF-50967603	Non-hospitalized person	A2	52	2138	2015	Troms	Faeces	No

Colour scheme correspond to sequence types as visualized in Fig. 6.

*Other infection site than blood, urine and faeces.

### Species diversity and genomic variations within bivalve samples

Among the 98 positive batch samples from 2019 and 2020 (prepared by a method allowing up to 15 isolates from each sample), 33 harboured more than one species of *Enterococcus *. Two different species were found in 29 batch samples, and the most common combination was *E. faecium* and *E. hirae* (Table S1). Eighteen batch samples of bivalves had also several *E. faecium* isolates within the same sample, and a great genetic diversity was found among these (Table 3).

**Table 3.** Overview of sequenced *E. faecium* isolates collected from the same batch sample of marine bivalves

Batch sample	County	Bivalve species	MPN/100 g	No. of *E.faecium* isolates	Sequenced isolates no.	ST	Cluster	Clade	Phenotypic AMR
2016–1129	Nordland	Blue mussels	340	4	1	296	426	B	None
					2	32	3613	A2	CIP, LEV
2019–2186	Trøndelag	Blue mussels	na	2	1	22	6063	A2	None
					2	328	6059	B	None
2020–1129	Trøndelag	Blue mussels	340	4	3	1046	6069	A2	None
					6	2173	7010	A2	None
					8	239	7012	B	Q/D
2020–113		Great scallops	45	2	2	Unknown	7008	A2	None
		Great scallops			3	Unknown	7008	A2	None
2020–312	Trøndelag	Blue mussels	220	3	1	52	2138	A2	None
					3	22	5173	A2	LEV
2020–324	Trøndelag	Blue mussels	490	7	1	2070	6091	A2	None
					2	437	6092	A2	None
					3	22	6063	A2	None
					4	328	6094	B	None
					5	1484	5611	B	Q/D
					6	623	6095	B	None
					7	361	6083	B	None
2020–331	Agder	Blue mussels	220	5	2	644	6068	A2	None
					3	22	6063	A2	None
					4	540	6099	B	None
					5	328	6098	B	CIP
					6	2075	6097	B	CIP
2020–374	Trøndelag	Blue mussels	78	2	1	1865	6100	A2	None
					3	94	7014	B	None
2020–375	Trøndelag	Blue mussels	130	4	1	94	6088	B	None
					2	994	7015	B	None
2020–533	Nordland	Blue mussels	490	6	1	581	6102	A2	None
					4	583	6089	B	None
					5	685	6077	B	CIP
					6	178	6084	B	CIP
					7	22	6063	A2	CIP
2020–635	Trøndelag	Blue mussels	170	3	1	22	6063	A2	None
					2	2175	7011	A2	None
2020–66	Nordland	Blue mussels	1300	3	2	361	1901	B	None
					9	159	7009	A2	None
2020–755	Trøndelag	Blue mussels	68	2	2	2077	6087	B	None
					3	117	2505	A1	AMC, AMX, AMP, GEN, STR, IMI
2020–975	Trøndelag	Blue mussels	45	2	1	2078	6107	A2	None
					2	2081	6108	A2	Q/D, STR
2020–989	Nordland	Blue mussels	3500	10	1	289	5243	B	None
					8	2078	6107	A2	IMI
2016–1063	Vestland	European flat oysters	<18	2	1	580	5402	B	CIP, LEV
					3	123	6065	B	CIP
2016–1167	Vestland	European flat oysters	790	2	1	646	7013	A2	CIP
					2	646	7013	A2	CIP, LEV
2020–311	Vestland	Great scallops	330	3	1	32	3613	A2	None
					3	361	1901	B	None
					4	361	1901	B	None

na – not analysed, CIP=ciprofloxacin, LEV=levofloxacin, Q/D=quinupristin/dalfopristin, AMC=amoxicillin-clauvulanic acid, AMX=amoxicillin, AMP=ampicillin, GEN=gentamicin, STR=streptomycin, IMI=imipenem.

## Discussion

### High prevalence, but low concentrations and low levels of antimicrobial resistance

This study found that marine bivalves frequently harbour enterococci (60.5 % prevalence, *n*=471), but in relative low concentrations where 49.5 % were at or below the LOQ at 18 MPN/100 g, and 86.2 % below 100 MPN/100 g. There was no correlation between the concentrations of enterococci and the concentrations of *E. coli* among the samples examined by quantitative methods (data not shown), which indicates that if originating from faecal sources, enterococci could sustain longer in the marine environment or originate from other sources independently from *E. coli *. Among the positive bivalve samples, more than half (56 %) contained *E. faecium *, which was the dominant identified species (Table 1).

### Where do the enterococci come from?

The ten enterococcal species found in the bivalves have mostly been reported associated with mammals and birds, but there are also occasional reports of aquatic hosts, mostly for *E. faecalis* and *E. faecium* [5]. *E. faecium* clade B has recently been reclassified as *E. lactis* [16]. It has been shown that MALDI-TOF BioTyper with in-house databases can differentiate between the *E. faecium* and *E. lactis* [44], but the current commercial databases used for MALDI-TOF do not distinguish between *E. faecium* and *E. lactis *. However, genome sequencing of a representative sample of 148 *E. faecium* revealed that 60 of these belonged to *E. lactis *, which have mainly been associated with human colonization, dairy products, probiotics and miscellaneous other food items [45]. In the global tree, the *E. faecium* from bivalves cluster with isolates derived from different isolation sources, such as humans, mammals and foods, but only three of the samples belonged to the typical hospital-associated clade A1 (Fig. 4). Some of the *E. faecium* strains from bivalves showed relatedness to Norwegian human samples isolated from the general population and clinical samples (Table 2 and Fig. 6). There are currently no *E. faecium* genomes from animal samples, food samples or wastewater samples collected in Norway available in the public databases. However, several of the STs found in the bivalve samples have been reported from other countries, including animals or food (ST10, ST12, ST22, ST29, ST32, ST70, ST123, ST159, ST218, ST437) [46] and wastewater (ST22, ST32, ST94, ST178, ST214, ST296, ST361, ST623, ST640, ST834, ST1205 and ST1206) [47]. These findings suggest that enterococci in the marine environment have diverse origins, reaching the ocean from different sources. Factors such as proximity to sewage outlets, urban areas and fertilized farmland, as well as tidal currents and time of sampling, are likely to influence both the concentrations and the prevailing strains. Lunestad *et al*. [10] showed that also the level of precipitation could influence the concentration of faecal bacteria in bivalves. Nevertheless, it is likely that most of the *E. faecium* isolated from bivalves along the Norwegian coast derive from healthy humans and animals.

### Expanding the *E. faecium* phylogenetic tree

Interestingly, the 148 *E. faecium* isolates that were selected for WGS, were almost equally divided between clades where 88 belonged to clade A and 60 to clade B (* E. lactis *) with broad genome sequence diversity within the clades (Fig. 5). Genome sizes did not vary significantly between isolates in either clade, nor did the effect of seasonality show any clear pattern. There is a large knowledge gap about population structure of *E. faecium* in natural waters (ocean, rivers, lakes). The five previously reported genomes of *E. faecium* from marine sources are an ST17 with a multidrug resistance plasmid from sediments of an Italian beach [18], an ST1336 with vancomycin resistance from brown mussels from the coastal shores of Brazil [21], and three linezolid-resistant isolates (two ST1710 and one ST1711) from sediments of the Italian coast [17]. The *E. faecium* of the Norwegian coastline were mostly susceptible to the tested antimicrobials, and none were resistant to vancomycin or linezolid. Thus, our collection of *E. faecium* that were representatively selected for sequencing, provides a large contribution to the non-clinical *E. faecium* database and to our understanding of *E. faecium* from the marine coastal environment. It is also noteworthy that these samples are collected from the coastline of a country with low frequency of resistance to vancomycin, and that it would be interesting to examine samples from the coastline of countries with higher prevalence.

### *E. faecium* from the same sample are genetically different

Not only different enterococcal species were found within the same batch samples of bivalves (Table 1), but also genetically different *E. faecium* (Table 3). Eighteen batch samples, each containing multiple *E. faecium* isolates showed large diversity, and only in one case did all the isolates originating from the same sample belong to the same sequence type. One of the batch samples contained as much as seven different sequence types. These isolates also differed in which clade they belonged to, the presence of plasmid replicons and in their antimicrobial resistance profile, supporting multiple sources of the isolates (Tables S2 and S4; Fig. 5). Each bivalve individual in a sample is collected at the same time, place and water level, and during method preparation they are combined and examined as one. The variations among the *E. faecium* may thus be explained by the composition of the water masses passing by the sampling area, but also the particle retention capacity of each individual bivalve. It is impossible to know whether one bivalve retained several different sequence types, or if the bivalves contributed with one sequence type each. But it is evident that the marine environment receives enterococci from multiple sources with different enterococcal species and sequence types. This is important to encompass when designing surveillance strategies targeting any key indicator bacteria in marine bivalves, where one isolate per sample [48] or several isolates from the same enrichment broth [49] represent the background data from where, prevalence is concluded. Examining isolates in parallel enrichments from the same sample, as done in this study, increases the resolution of both the phylogeny and prevalence of resistance, as well as providing quantitative data on enterococci. Qualitative methods with selective enrichment have shown better sensitivity (lower limit of detection) than quantitative methods [50], however, any enrichment prior to plating will make it difficult to assess the inter- and intra-species diversity. The bivalves are likely to accumulate the different strains, whereas it is the downstream analyses that limit the resolution of the findings.

## Conclusion

This study reports high prevalence but relatively low concentrations of enterococci in marine bivalves harvested along the Norwegian coast. Among the 247 examined *E. faecium* isolates, only five (2 %) were resistant to three or more antimicrobial classes, and three of these belong to typical hospital associated clones. The majority of examined isolates resembled strains rarely involved in human infections, indicating a possible, but low risk for foodborne enterococcal infection vehicled by bivalves from the examined areas. Marine bivalves are good indicator tools for monitoring the level of antimicrobial resistance in the marine environment. However, care should be taken when designing downstream analysis as we here demonstrated high diversity among enterococci from the same batch samples. This study provides evidence that enterococci reach the ocean through multiple sources and are not only linked to sewage pollution.

## Supplementary Data

Supplementary material 1

Supplementary material 2
