# Supplementary material for: Genomic insights into Enterococcus faecium isolates from marine bivalves highlight One Health concerns and healthcare linkages
Source: Microb Genom. 2023 Dec 12;9(12):001154. doi: 10.1099/mgen.0.001154 (PMC10763502; doi:10.1099/mgen.0.001154)
Supplement: Supplementary material 2 [file mgen-9-1154-s002.pdf]

Supplementary Table 3. Distribution (n) of MIC values (mg/L) for *E. faecium* bacterial isolates (n=247) and the tested antimicrobial agents. Breakpoint values are based on the EUCAST Clinical Breakpoint Tables v.12.

***E. faecium* MIC distribution**	**0.03**	**0.06**	**0.125**	**0.25**	**0.5**	**1**	**2**	**4**	**8**	**16**	**32**	**64**	**128**	**256**	**512**	**1024**	**Total**	
Ampicillin				41	53	99	47	4			3						247	
Amoxicillin				83	118	39	4				3						247	
Amoxicillin / clavulanic acid				72	116	52	4				3						247	
Vancomycin					114	102	26	5									247	
Teicoplanin					242	5											247	
Trimethoprim*	3	7	44	120	49	5		19									247	
Linezolid					1	122	119	5									247	
Nitrofurantoin											3	244					247	
Streptomycin															238	9	247	
Gentamicin											245			2			247	
Imipenem					18	33	44	141	4	7							247	
Q/D (only *E. faecium*)				1	93	19	111	18	5								247	
Tigecycline		136	96	14			1										247	
Ciprofloxacin					13	62	69	66	24	13							247	
Levofloxacin					4	28	94	99	11	11							247	

**No breakpoint for trimethoprim.*

Supplementary Table 5. Samples that contained more than one *Enterococcus* species.

Sample ID	Year	From bivalve species	No. isolates/ sample	No. of different species
2020-855	2020	Blue mussel	13	*9 E. faecium, 4 E. durans*
2020-989	2020	Blue mussel	11	*10 E. faecium, 1 E.durans*
2020-66	2020	Blue mussel	9	*3 E. faecium, 6 E. hirae*
2020-1129	2020	Blue mussel	8	*4 E. faecium, 1 E. faecalis, 2 E. hirae, 1 E. mundtii*
2020-331	2020	Blue mussel	7	*5 E. faecium, 1 E. faecalis, 1 E. hirae*
2020-237	2020	Blue mussel	7	*4 E. faecium, 3 E. hirae*
2020-312	2020	Blue mussel	6	*3 E. faecium, 3 E. hirae*
2020-635	2020	Blue mussel	5	*3 E. faecium, 2 E. hirae*
2020-1124	2020	Blue mussel	5	*4 E. faecalis, 1 E. durans*
2020-634	2020	Blue mussel	5	*4 E. hirae, 1 E.faecium*
2020-317	2020	European flat oyster	5	*4 E. faecium, 1 E. hirae*
2020-274	2020	Blue mussel	5	*4 E. faecium, 1 E. faecalis*
2020-1126	2020	Blue mussel	4	*2 E. faecalis, 1 E. faecium, 1 E. thailandicus*
2020-1123	2020	Blue mussel	4	*2 E. durans, 1 E. faecium, 1 E. hirae*
2020-756	2020	Blue mussel	3	*1 E. faecium, 1 E.hirae*
2020-1013	2020	Blue mussel	3	*2 E. thailandicus, 1 E. casseliflavus*
2020-374	2020	Blue mussel	3	*2 E. faecium, 1 E. hirae*
2020-32	2020	Blue mussel	3	*2 E. faecium, 1 E. faecalis*
2020-755	2020	Blue mussel	3	*2 E. faecium, 1 E.hirae*
2016-1063	2016	European flat oyster	3	*2 E. faecium, 1 E. hirae*
2020-113	2020	Great scallop	3	*2 E. faecium, 1 E. hirae*
2020-983	2020	Blue mussel	3	*2 E. faecalis, 1 E. mundtii*
2016-1064	2016	Blue mussel	2	*1 E. mundtii, 1 E. hirae*
2016-1199	2016	Blue mussel	2	*1 E. faecium, 1 E. faecalis*
2020-275	2020	Blue mussel	2	*1 E. hirae, 1 E. faecalis*
2020-521	2020	Blue mussel	2	*1 E. faecium, 1 E. hirae*
2016-1198	2016	Blue mussel	2	*1 E. faecium, 1 E. hirae*
2020-519	2020	Blue mussel	2	*1 E. faecium, 1 E. hirae*
2016-1172	2016	Blue mussel	2	*1 E. faecium, 1 E. hirae*
2020-671	2020	Blue mussel	2	*1 E. faecalis, 1 E. faecium*
2020-230	2020	Blue mussel	2	*1 E. hirae, 1 E. casseliflavus*
2020-235	2020	Blue mussel	2	*1 E. faecalis, 1 E. faecium*
2016-945	2016	Blue mussel	2	*1 E. faecalis, 1 E. hirae*
